# Uncommon Malposition of an Ultrasound-Guided Central Venous Catheter in the Renal Vein through the Superficial Femoral Vein: A Case Report

**DOI:** 10.2478/jccm-2024-0026

**Published:** 2024-07-31

**Authors:** Ting-Chia Remus Young, Kuang-Hua Cheng, Kuan-Pen Yu

**Affiliations:** Department of Critical Care Medicine, MacKay Memorial Hospital, Taipei, Taiwan; Department of Medicine, Mackay Medical College, New Taipei, Taiwan; Graduate Institute of Clinical Medicine, College of Medicine, National Taiwan University, Taiwan; Department of Anesthesiology, MacKay Memorial Hospital, Taipei, Taiwan

**Keywords:** peripheral catheterization, femoral vein, renal veins, adverse effects, intensive care

## Abstract

**Introduction:**

Malposition is a relatively rare complication associated with peripherally inserted central catheters (PICCs), particularly in cases of superficial femoral vein (SFV) catheterization. To the best of our knowledge, we are the first to report this rare case of SFV PICC malposition in the contralateral renal vein.

**Case presentation:**

An 82-year-old woman underwent bedside cannulation of the SFV for PICC under ultrasound guidance. Subsequent radiographic examination revealed an unexpected misplacement, with the catheter tip positioned toward the contralateral renal vein. After pulling out the catheter on the basis of the X-ray result, it was observed that the catheter retained its function.

**Conclusion:**

Although rare, tip misplacement should be considered in SFV PICC placement. Prompt correction of the tip position is crucial to prevent catheter malfunction and further catastrophic consequences. For critical patients receiving bedside SFV PICC insertion, postoperational X-ray is crucial for enhancing safety.

## Introduction

Patients with superior vena cava (SVC) syndrome are contraindicated to undergo placement of upper limb peripherally inserted central catheters (upper limb PICCs), and cannulation of veins in the lower limb is required [1]. PICCs introduced via the superficial femoral vein at the midthigh (SFV PICCs) are advanced strategies developed after traditional common femoral vein catheterization with the puncture site placed over the groin area (CFV PICCs). SFV PICCs are associated with a lower risk of infection and thrombotic complications than CFV PICCs [2]. Malposition of SFV PICCs, where the catheter tip is not in the main trunk of the inferior vena cava (IVC), is a rare adverse event with limited reports. Therefore, we have described a case of SFV PICC tip malposition in the contralateral renal vein.

## Case Presentation

An 82-year-old woman, without any history of systemic disease, was presented to the emergency department owing to episodes of vomiting for 1 week. She also reported constipation for several days. With a preliminary diagnosis of ecchymosis of the ileum with incarceration into the obturator foramen, she was hospitalized for an emergency operation. Laparoscopic repair of the inguinal hernia with segmental resection of the small bowel of approximately 6 cm with side-to-side anastomosis was then performed.

Postoperatively, the patient was admitted to the intensive care unit (ICU). Owing to the pronounced ecchymosis and edema observed over both upper limbs, an SFV PICC (ARROW^®^, 5 Fr, two lumens, 55-cm length, Teleflex Incorporated) was introduced into the right SFV by an experienced anesthesiologist for intravenous access of total parenteral nutrition,. Given the patient’s unstable medical condition, the procedure was performed at the patient’s bedside under ultrasound guidance. The diameter of the right SFV was approximately 8 mm, and the depth was 3 cm from the skin under ultrasound visualization. The right midthigh area, which is 15 cm away from the inguinal ligament, was selected as the best site for cannulation of the right SFV. The right SFV was visualized in short-axis, out-of-plane technique at an angle of 45°. Using a modified Seldinger technique, the right SFV was percutaneously accessed, followed by the advancement of a metallic guidewire. The sheath dilator was then threaded through the guidewire before the catheter was positioned within the vein at a total depth of 55 cm. The initial insertion was smooth, and the entire procedure was completed in 10 min. Normal saline irrigation was performed on the catheter to verify whether it was functioning properly. The exit site was covered with a 3M™ Tegaderm™. The entire process of cannulation was conducted in accordance with the Practice Guidelines for Central Venous Access 2020 by the American Society of Anesthesiologists [3].

**Fig. 1.**
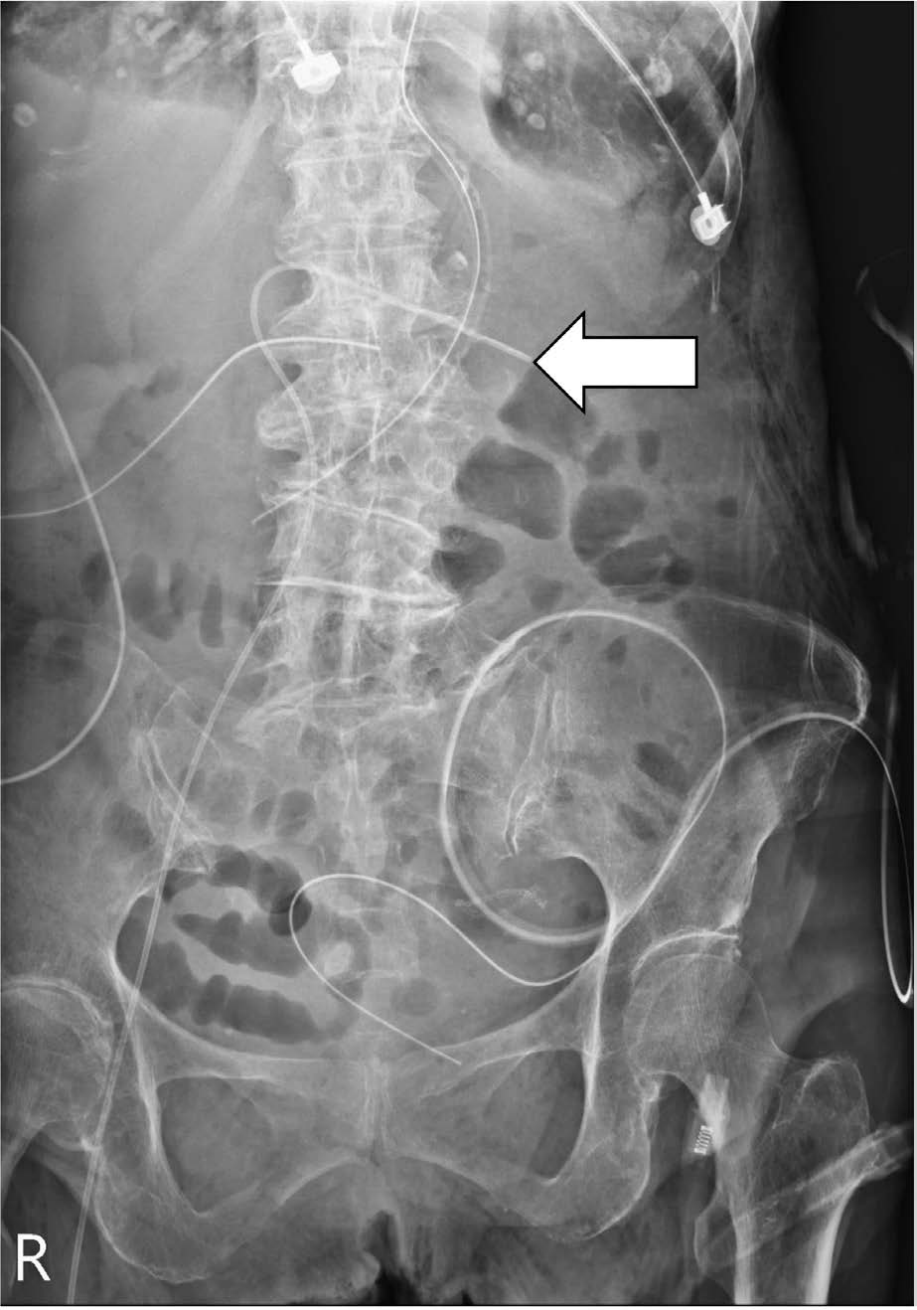
Postprocedural abdominal X-ray showing misplacement of the catheter tip in the contralateral renal vein (arrow).

**Fig. 2.**
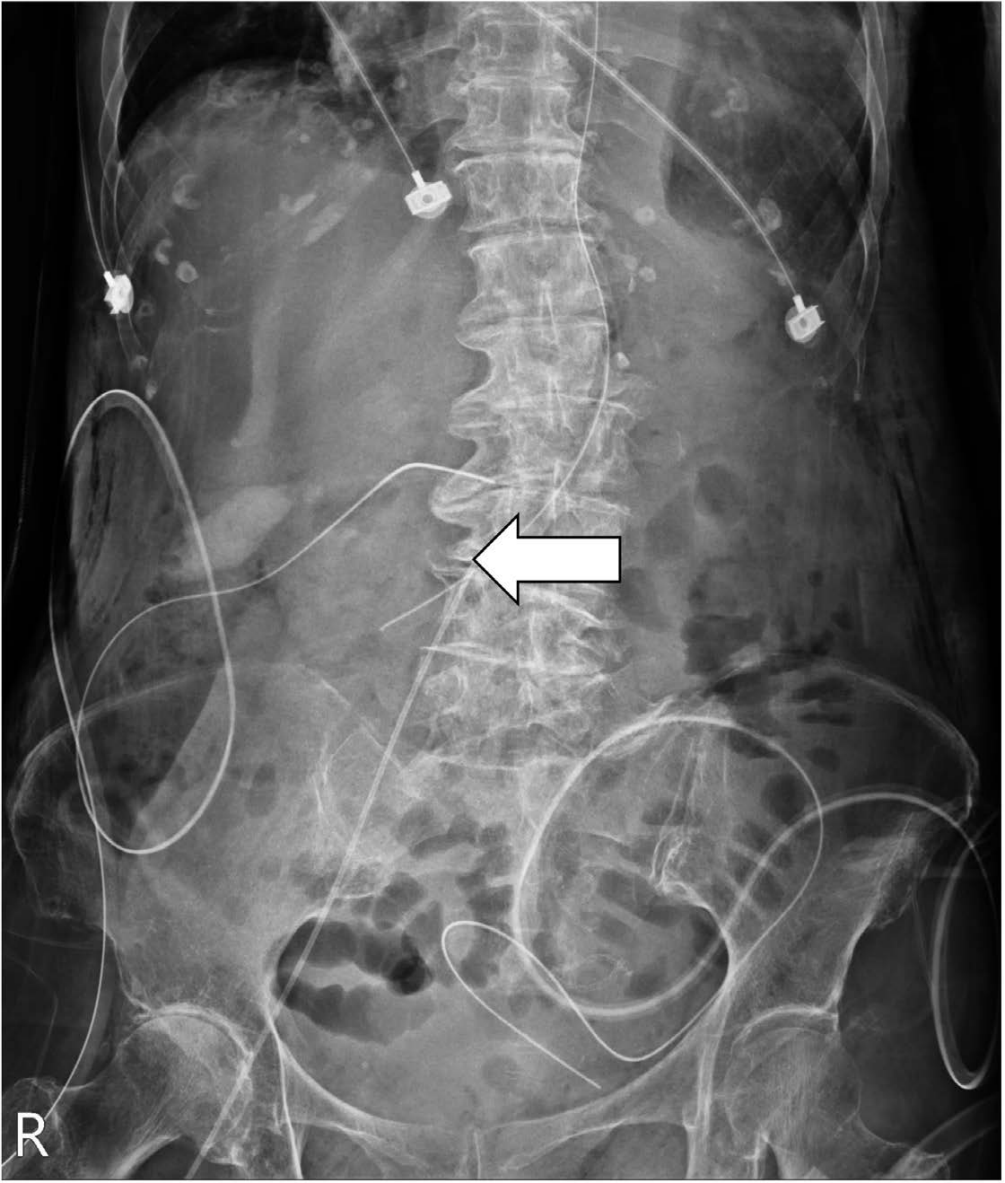
Relocation of the catheter tip within the middle segment of the inferior vena cava after pulling out by 8 cm (arrow).

Postprocedural abdominal radiography revealed inadvertent mispositioning of the catheter into the contralateral renal vein (Figure 1). We pulled the catheter out by 8 cm based on the radiological findings (Figure 2). The SFV PICC was used on admission to the ICU and general ward, and no bleeding, infection, or other complications were noted during the entire hospital course. Computed tomography before the surgery and abdominal sonography during the follow-up period revealed no evidence of vein thrombosis. The patient’s clinical status improved with proper nutrition through SFV PICC and adequate wound management. The removal of the SFV PICC was performed at the time of the patient’s discharge on Day 20.

## Discussion

A rare malposition of an SFV PICC into the contralateral renal vein was reported in this report. This malposition was observed in post procedural abdominal X-ray and managed successfully by withdrawing the catheter by 8 cm. The patient did not have any end-organ damage.

SFV cannulation may reduce the rate of bloodstream infection and thrombosis historically seen in patients with CFV PICC implantation [4]. According to a single-center cohort study by Zhang et al., which investigated the overall benefits among patients with lower extremity PICCs (SFV/CFV PICCs) through different insertion sites, it was suggested that placing the entry point in the SFV around the midthigh region can improve patient comfort, extend catheter retention duration, and reduce complications and pain scores. Meanwhile, the success rate of the first attempt of venipuncture did not decrease [5]. Hence, it is possible that the reduction of hair and moisture at the midthigh area contributes to a lower risk of bloodstream infections. Besides, the SFV PICCs dressing can be easily managed and observed as compared with CFV PICCs [6]. The patient in this study developed upper limb swelling and bruising after the operation, making it impractical to place an upper limb PICC. Additionally, the lower limb vessels were comparatively larger, prompting us to opt for SFV PICC placement.

SFV PICCs have a very low rate of adverse events, with cases of misplacement being even rarer [2]. We identified three retrospective cohort studies documenting a collective total of six patients who experienced primary malposition after SFV PICC insertion (Table 1). Wan et al. and Zhao et al. investigated SFV PICCs in patients with SVC syndrome mediastinal tumors and indicated a lower risk of malposition as compared with upper limb PICCs. This may be related to the abundance of branches within the upper extremities, whereas the femoral vein follows a more direct path without bifurcations. In a retrospective cohort study by Zhao et al., two patients had primary malposition [7,8]. An 89-year-old woman who received antimicrobial and low-concentration nutrient treatment exhibited primary malposition of the catheter toward the contralateral common iliac vein. In another case, the catheter of a 73-year-old man was folded in the common iliac vein. Although the abovementioned two cases are the only detailed documentation of SFV malposition that has been published and were both confined in the common iliac vein, our case suggests the potential for malposition into a narrower vessel draining into the IVC. A cross-sectional study by Elli et al. revealed that the risk of complications decreased considerably when placing the exit site at the midthigh area rather than at the groin; however, 4 of 142 patients still experienced primary malposition [9]. Additionally, Josiak et al. reported a case of PICC malposition into the right renal vein, which is the only previous study with a similar malposition site as our case [10]; however, the exit site of the PICC was not specified in that case. Our case resembles those reported previously that primary malposition of an SFV PICC might be rare, but it still exists and has potential complications.

**Table 1.** Previously reported malposition cases with SFV PICCs

**First author (year)**	**Number of patients**	**Number of malposition cases (%)**	**Age/Sex**	**Malposition place**	**Subsequent management**	**Result of interest**
Wan (2018)	221	0 (0)	n/a	n/a	n/a	Significant lower malposition rate in patients with SFV PICCs (0%) compared to upper limb PICCs (2.15%).

Zhao (2019)	121	2 (1.65)	89/f	Contralateral common iliac vein	No adjustment or re-puncture based on the decision from the doctor and the patient’s guardian.	Significant lower malposition rate in patients with SFV PICCs (1.65%) compared to upper limb PICCs (6.02%).
			73/m	Folded at ipsilateral common iliac vein	Pull out 4 cm according to the X-ray findings.	

Elli (2022)	142	4 (2.82)	n/a	n/a	Immediate replacement	n/a

Although anatomical variations in the IVC and femoral vein are common, the causes of malposition are multifactorial, including operator experience, insertion technique, and patient status. Richter et al. used ultrasound to confirm the SVF PICC tip position in critically ill infants; however, its applicability in adults remains unknown [11]. Intracavitary electrocardiogram is deemed the gold standard for the confirmation of upper limb PICC tip position by observing P-wave amplitude changes near the SVC/right atrium (SVC/RA) junction; however, unlike upper limb PICCs extending to the SVC/RA junction, most SFV/CFV PICCs are placed in the middle segment of the IVC. Bedside placement of PICC is crucial for critical patients to avoid the need to transport the patient; therefore, continuous fluoroscopy guidance is not feasible. In such situations, postprocedural X-ray to confirm the location of the tip becomes even more crucial, especially when tip adjustment is easily manageable.

Although SFV/CFV PICC malposition has seldom been documented, misplaced upper limb PICCs increase the risks of deep vein thrombosis, vessel perforation, arrhythmia, and tamponade [12]. In this case, we promptly inserted a SFV PICC within 24 h of surgery to meet the patients’ nutritional requirements. Because the patient had preexisting catheters and was at a high postoperative risk, it was inappropriate to transport her for fluoroscopy. Despite slightly higher chance of tip readjustment compared with fluoroscopy, considering the inherently low chances of tip malposition of SFV PICCs, bedside placement followed by X-ray checkup confirms proper tip location and avoids unnecessary patient movement.

## Conclusion

In conclusion, although often underreported in the literature, the primary malposition of SFV PICCs must be considered during placement. In this study, the malposition was instantly corrected to help avoid catheter malfunction and she was successfully discharged after nutrition support through the SFV PICC. During the follow-up period, the patient showed no signs of malposition complications such as deep vein thrombosis and vessel perforation. Ultimately, SFV PICC, with a focus on tip position correction, remains a preferable choice for vascular access because of its relatively low complication rate. Postplacement X-ray enhances safety, particularly in critically ill patients undergoing bedside-inserted SFV PICCs.
